# Axon Branch-Specific Semaphorin-1a Signaling in *Drosophila* Mushroom Body Development

**DOI:** 10.3389/fncel.2016.00210

**Published:** 2016-09-05

**Authors:** Liesbeth Zwarts, Tim Goossens, Jason Clements, Yuan Y. Kang, Patrick Callaerts

**Affiliations:** ^1^Laboratory of Behavioral and Developmental Genetics, Department of Human Genetics, KU Leuven, LeuvenBelgium; ^2^Center for the Biology of Disease, Vlaams Instituut voor Biotechnologie, LeuvenBelgium; ^3^Department of Biology and Biochemistry, University of Houston, Houston, TXUSA

**Keywords:** mushroom body, *Drosophila*, Semaphorin-1a, development, axon branch-specificity

## Abstract

Correct wiring of the mushroom body (MB) neuropil in the *Drosophila* brain involves appropriate positioning of different axonal lobes, as well as the sister branches that develop from individual axons. This positioning requires the integration of various guidance cues provided by different cell types, which help the axons find their final positions within the neuropil. Semaphorins are well-known for their conserved roles in neuronal development and axon guidance. We investigated the role of Sema-1a in MB development more closely. We show that Sema-1a is expressed in the MBs as well as surrounding structures, including the glial transient interhemispheric fibrous ring, throughout development. By loss- and gain-of-function experiments, we show that the MB axons display lobe and sister branch-specific Sema-1a signaling, which controls different aspects of axon outgrowth and guidance. Furthermore, we demonstrate that these effects are modulated by the integration of MB intrinsic and extrinsic Sema-1a signaling pathways involving PlexA and PlexB. Finally, we also show a role for neuronal- glial interaction in Sema-1a dependent β-lobe outgrowth.

## Introduction

Due to its characteristic structure, the mushroom body (MB) neuropil forms a powerful model system to dissect the molecular cues underlying axonal guidance. The MBs derive from four neuroblasts per brain hemisphere, which give rise to a total of about 2500 intrinsic MB cells called Kenyon cells. The MB axons project ventromedially through the peduncle and then branch to form different cell type specific lobes (Crittenden et al., 1998; Lee et al., 1999). In larvae, γ neurons form a vertical and a medial axonal lobe. During metamorphosis, these lobes are pruned and a single medial γ lobe is established. The later born α′β′ and αβ neurons form sister branches that then project into the vertical α and α′ lobes and the horizontal β and β′ lobes (Lee et al., 1999). Considerable effort has been made to provide insights into the development of this neuropil. However, very little is still known about the guidance cues underlying lobe or even sister branch-specific development.

In this study, we focused on the role of Semaphorin-1a (Sema-1a) in MB development. The Sema-1a protein is a member of the Semaphorin family, a group of axon guidance molecules well-known for their role in axon guidance in vertebrates as well as in invertebrates. This family, subdivided into eight subfamilies of secreted and membrane bound molecules, is characterized by a 500 amino acid extracellular Sema domain. Neuropilins and Plexins have been identified as the two main families of Semaphorin receptors. In *Drosophila*, five Semaphorin gene family members and two Plexins have been identified, while no Neuropilin homologs were found (Kruger et al., 2005).

*Drosophila* Sema-1a has been shown to mediate embryonic motor and CNS axon guidance and to control axon guidance and synapse formation in the giant fiber system (Yu et al., 1998; Godenschwege et al., 2002). Sema-1a also functions as a guidance receptor in the visual system and the olfactory projection neurons, while it acts as a ligand in axonal guidance of olfactory receptor neurons (Cafferty et al., 2006; Lattemann et al., 2007; Sweeney et al., 2007). Finally, it has also been shown to modulate α′β′ lobe development in the MBs (Komiyama et al., 2007).

We investigated the role of Sema-1a in MB development more closely. We show that *Sema-1a* is expressed in the MBs as well as surrounding structures, including the glial transient interhemispheric fibrous ring (TIFR), throughout development. By loss- and gain-of-function experiments we show that the MB axons display lobe and sister branch-specific Sema-1a signaling, which controls aspects of axon outgrowth and guidance. We demonstrate that these effects involve MB intrinsic and MB extrinsic Sema-1a signaling pathways. Furthermore, we confirm a role for PlexinA (PlexA) as a Sema-1a receptor, but also provide evidence of a genetic interaction between Sema-1a and PlexinB (PlexB). Finally, we show a role for neuronal- glial interactions in Sema-1a dependent β-lobe outgrowth.

## Materials and Methods

### *Drosophila* Stocks

Flies were reared at 25°C on standard *Drosophila* yeast-cornmeal molasses media. The following stocks were used: *OK107-Gal4*, *Sema-1a^k13702^*, *Df(2l)Exel7039*, *PlexB^KG00878^*, *PlexA^EY 16548^*, UAS-*lacZ.btau.YES*, UAS*-mCD8-Gfp*, UAS*-mCD8-Rfp* (Bloomington stock center, Bloomington, IN, USA), *Sema-1a^CA7125^* and *PlexA^Y D0269^* (A. Spradling, Carnegie Institution for Science, Baltimore, MD, USA), 442-*Gal4* (T. Préat, Ecole Supérieure de Physique et Chimie Industrielle, Paris, France), UAS-*Sema-1a*-RNAi, UAS*-PlexB*-RNAi and UAS-*PlexA*-RNAi (L. Luo, Stanford University, Stanford, CA, USA; The efficacy and specificity of these RNAi constructs has been previously reported (Hu et al., 2001; Sweeney et al., 2007; Yu et al., 2010), UAS-*PlexB* and UAS-*Sema-1a* (C. Goodman, University of California, Berkeley, CA, USA), UAS-*Sema-1a^Δcyt1^* (T. Godenschwege, University of Massachusetts, Amherst, MA, USA), UAS-*Sema-1a^Δcyt2^* (A. Kolodkin, Johns Hopkins University, Baltimore, MD, USA). For mosaic analysis with a repressible cell marker (MARCM) analyses, *hsFLP*; *Sema-1a^k13702^ FRT40a*/*FRT40A-Gal80; Tub-Gal4*, UAS*-Gfp-cd8* flies were heat shocked at 37°C for 1 h at the relevant developmental stage (Lee et al., 1999).

### *In situ* Hybridization

cDNA clones for *PlexA* (LD10519) and *PlexB* (RE22882) were ordered from the Berkeley *Drosophila* Genome Project. *In situ* hybridization was done as previously described (Edwards et al., 2009). Images were obtained using a light microscope (model BX61; Olympus) and CellˆD 2.6 imaging software.

### Immunohistochemistry

Immunohistochemical labeling of *Drosophila* brains was done as previously described (Rollmann et al., 2008). The following antibody dilutions were used: monoclonal 1D4 antibody (anti-FasII): 1:100; 4F3 antibody (anti-Dlg1): 1:20 (Developmental Studies Hybridoma Bank, University of Iowa, Iowa City, IA 52242, USA); anti-GFP: 1:500 (Abcam, Cambridge, UK); anti-Sema-1a: 1:5000 (A. Kolodkin, Johns Hopkins University, Baltimore, MD, USA). FITC- or Cy3-labeled anti-mouse or anti-rabbit antibodies (1:200; Jackson Immunoresearch, Westgrove, PA, USA). Confocal imaging was performed using an Olympus FV1000 microscope. Defects in lobe length and orientation were defined by visually inspecting all brains. Only obvious and unambiguous differences in length and/or orientation were considered.

### Statistical Analyses

Statistical analyses were performed using Graphpad Prism 6. Statistical tests were chosen based on the format of the data (continuous or ordinal) and the distribution (Gaussian).

## Results

### *Sema-1a* is Expressed in the Mushroom Bodies during Development as Well as in Adults

**FIGURE 1:**
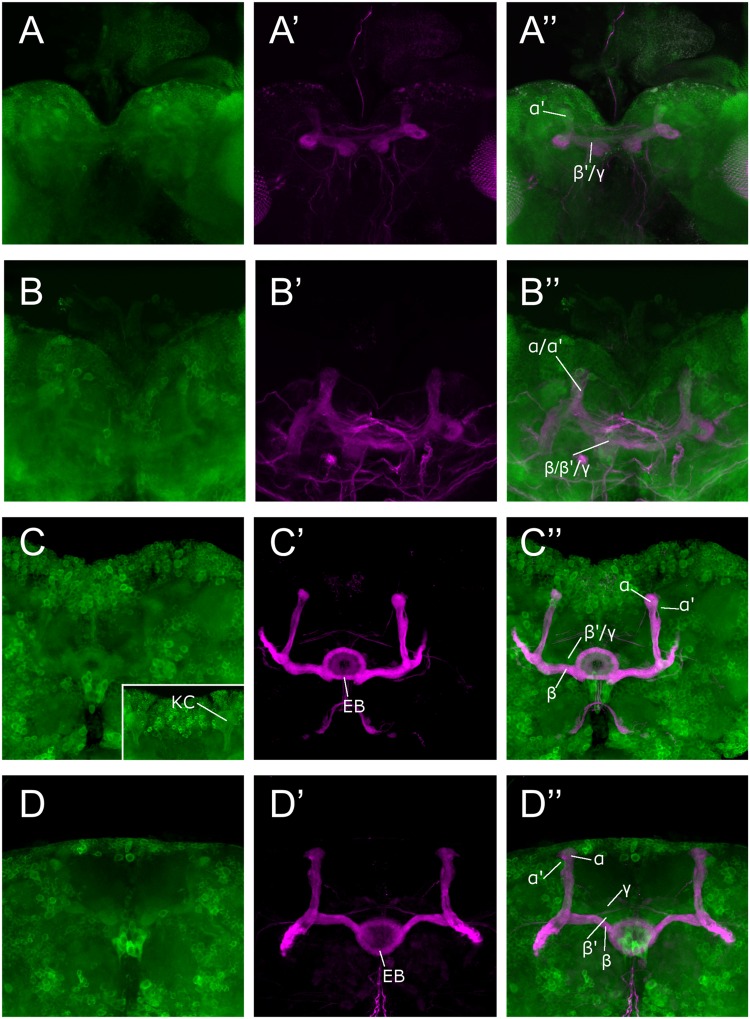
**Sema-1a expression analysis.** Anti-GFP labeling (green) of the *Sema-1a^CA07125^* GFP protein trap line combined with anti-FasII labeling (magenta) visualizing the α, β, and γ lobes of the MBs and the ellipsoid body (EB; Fushima and Tsujimura, 2007). Sema-1a is expressed in cell bodies throughout the cortex of the larval and adult brain as well in as axonal tracts such as the larval MBs and the adult EB and MB. We detected labeling in all the MB lobes of third instar larvae as well as during all pupal stages. **(A)** Wandering third instar larvae: anti-FasII labels the γ-lobes, other GFP positive MB lobes consist of the developing α′ and β′ lobes **(B)** 25% pupae: anti-FasII labels α, β and weakly the γ lobes, other GFP positive MB lobes consist of the developing α′ and β′ lobes. **(C)** 50% pupae: anti-FasII labels α, β and weakly the γ lobes, other GFP positive MB lobes consist of the developing α′ and β′ lobes. We also observe GFP expression in the MB Kenyon cells (KC; inset). **(D)** 75% pupae: anti-FasII labels α, β, and γ lobes, other GFP positive MB lobes consist of the developing α′ and β′ lobes.

We used the *Sema-1a^CA07125^* GFP protein trap line to study the expression pattern of *Sema-1a* in the MBs. This line contains a *P{PTT-GA}* insertion between the first two exons of the gene, generating a GFP::Sema-1a fusion protein (Buszczak et al., 2007). We observe prominent expression in cell bodies throughout the cortex of the larval and adult brain as well in as axonal tracts such as the larval MBs and the adult ellipsoid body (EB) and MBs. We detected labeling in all the MB lobes of third instar larvae as well as during all pupal stages (**Figure 1**). We confirmed this MB expression using an antibody targeting the Sema-1a protein (Supplementary Figures S1A–C). Expression of *Sema-1a* in the MBs persisted throughout adulthood (Supplementary Figure S1D).

### *Sema-1a* Regulates MB Lobe Length and Orientation

We showed that *Sema-1a* is expressed in the MBs during different developmental stages. To investigate the role of Sema-1a in MB development we made use of loss- and gain-of-function experiments. For these analyses, we focused on the α, β, and γ lobes as these are easy to visualize and showed the most prominent phenotypes. We also documented the effects of Sema-1a on α′ and β′ lobe development (Supplementary Tables S1–S3).

**FIGURE 2:**
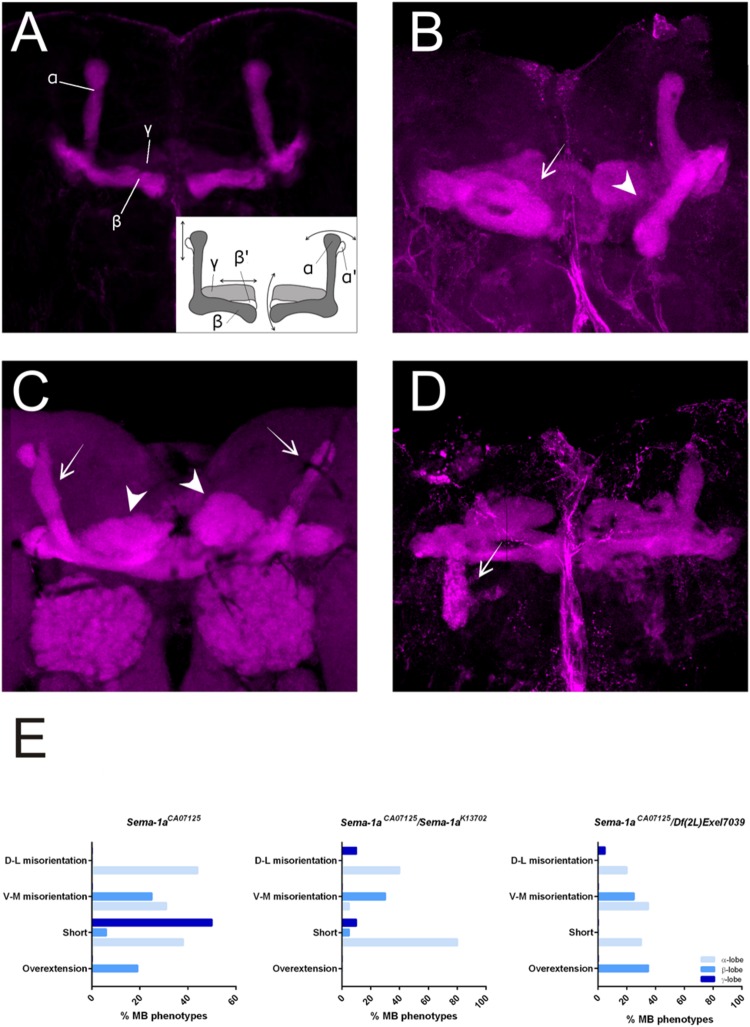
**Sema-1a mutants show MB defects. (A)** Anti-FasII staining of wild-type MBs labeling the α, β and more weakly the γ lobes. Inset: overview of the two categories of observed phenotypes. Left side of the scheme represents defects in lobe outgrowth, resulting in overextended or short lobes. Right side represents orientation defects, resulting in an abnormal angle between the vertical and horizontal lobes. (Increased angle: vertical lobes growing more lateral, or horizontal lobes growing more ventral; Decreased angle: vertical lobes growing more medial, or horizontal lobes growing more dorsal, in the most extreme case resulting in β-to-α or α-to-β misguidance.) **(B)** *Sema-1a^CA07125^/Sema-1a^K13702^*: anti-FasII staining. Both α and β lobes show guidance defects. One α lobe is projected parallel to the β lobe (arrow). β lobes are projected more ventrally (arrow head). **(C,D)** *Sema-1a^CA07125^/Df(2l)Exel7039* flies. **(C)** Anti-Dlg1 staining, labeling all MB lobes. α lobes are projected laterally (arrows), β and γ lobes have an abnormal morphology, due to outgrowth and guidance defects (arrowheads). **(D)** Anti-FasII staining. One α lobe is projected ventrally (arrow). **(E)** Summary of the different MB lobe defects observed in *Sema-1a^CA07125^* homozygotes and *Sema-1a^K13702^/Sema-1a^CA07125^* or *Sema-1a^CA07125^*/*Df(2L)Exel7039* heterozygotes. [Dorsal- lateral (D-L) misorientation, ventral- medial (V-M) misorientation, short, overextension].

First, we made use of three mutant lines; a deficiency covering the *Sema-1a* locus and two P-element insertion lines, the null allele *Sema-1a^k13702^* and the hypomorphic *Sema-1a^CA07125^* allele (Yu et al., 1998; Buszczak et al., 2007). *Sema-1a^k13702^* contains a *PlacW* insertion located in the 5′ UTR of the *Sema-1a* gene (Yu et al., 1998). *Sema-1a^CA07125^* was described in Section “*Sema-1a* is Expressed in the Mushroom Bodies during Development as Well as in Adults.” These lines were all backcrossed into a Canton-S background to eliminate possible confounding effects due to genetic background. In the wild type Canton-S flies themselves, we did not observe any MB defects (*n* = 40; **Figure 2A**). Homozygous *Sema-1a^k13702^* flies do not survive beyond the first larval stage. Homozygous *Sema-1a^CA07125^* mutants are semi-lethal, with survivors showing strong MB defects (**Figure 2E**). The same range of phenotypes was seen in *trans*-heterozygous *Sema-1a^k13702^*/*Sema-1a^CA07125^* and *Sema-1a^k13702^*/*Df(2l)Exel7039* flies (**Figures 2A–C**). We observed two types of defects: alterations in lobe length, resulting in short or overextended lobes and alterations in lobe orientation, resulting in ventral-medial or dorsal-lateral misoriented lobes (**Figure 2A**).

### *Sema-1a* has MB Intrinsic and Extrinsic Effects on Lobe Length

**FIGURE 3:**
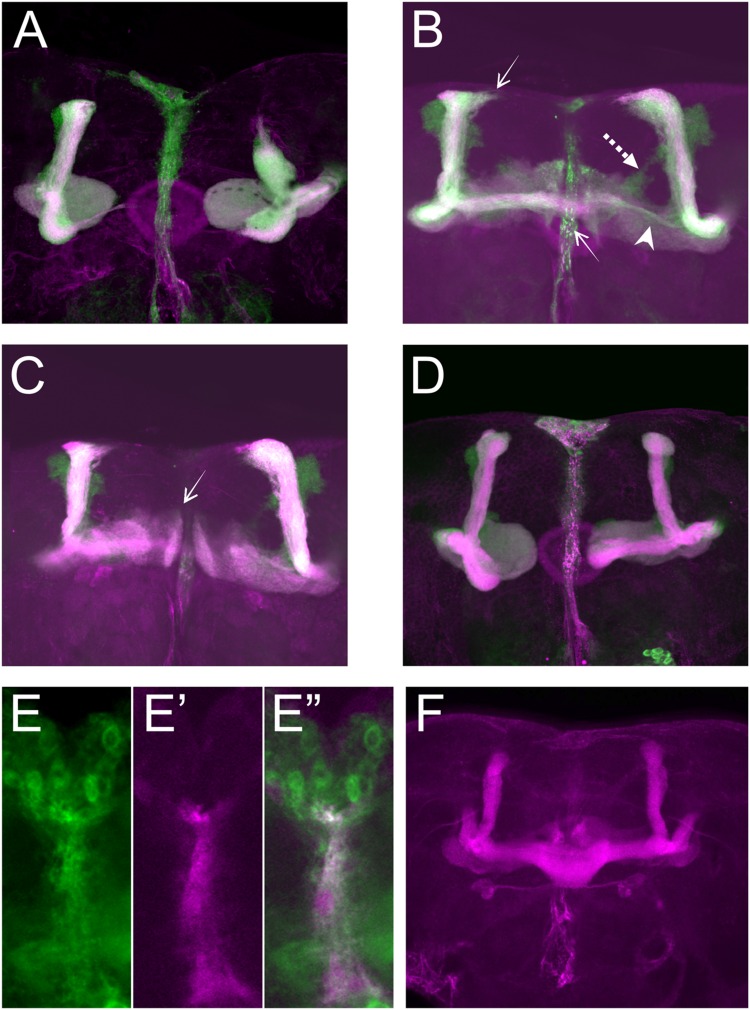
**Sema-1a MB intrinsic and extrinsic effects. (A–D)** *OK107-Gal4*-driven *UAS-mCD8-Gfp* labeling all MB lobes (green), Anti-FasII labeling α, β, and γ lobes (magenta). Confocal stacks **(A)** *UAS-RNAi-Sema-1a; UAS-mCD8-Gfp; OK107-Gal4:* MB lobes show outgrowth defects resulting in shorter α, β, γ, α′, and β′ lobes. **(B,C)** *UAS-Sema-1a; UAS-mCD8-Gfp; OK107-Gal4.* **(B)** α and β lobes show overextension (arrow). Most β axons show misorientation and project vertically besides the α lobe, resulting in a thin horizontal lobe (arrow head). α′β′ axons do not project properly and fuse (dashed arrow). **(C)** γ-lobes have abnormal morphology with tips growing up (arrow). **(D)** *UAS-Sema-1a^Δcyt2^, UAS-mCD8-Gfp;OK107-Gal4:* β and γ lobe show outgrowth defects resulting in shorter lobes. **(E,E′,E′′)** Frontal view of the pupal TIFR, 24 h after puparium formation. **(E)** *Sema-1a^CA07125^*: anti-GFP staining (green), Confocal stacks **(E′)** *442-Gal4/UAS-mCD8-Rfp*: RFP expression in the TIFR (magenta). **(E′′)** Overlay. **(F)** Anti-FasII labeling α, β, and γ lobes. *UAS-RNAi-Sema-1a; 442-Gal4*: knock-down of *Sema-1a* in the TIFR results in β lobe overextension and fusion.

For further analyses, we first focused on the alterations in lobe length. Loss of Sema-1a in the investigated mutants affected the length of the MB lobes in a lobe-specific manner. The axons of the α lobes were often short, while their axonal sister branches in the β lobes displayed overextension phenotypes (**Table 1**; **Figure 2**). The γ lobes were also short. To examine the MB intrinsic requirement for Sema-1a, we looked at the effects of *Sema-1a* RNAi-mediated gene knock-down and overexpression using the *OK107-Gal4* MB-driver (Sweeney et al., 2007; Yu et al., 2010). *OK107-Gal4* drives expression in all MB neuroblasts, ganglion mother cells and neurons from embryonic stages onward (Adachi et al., 2003). Knock-down of *Sema-1a* in the MB neurons resulted in short α and γ lobes, reminiscent of the phenotype observed in the mutants. In contrast with the mutant lines, the β lobe phenotype shifted from overextension to shorter lobes (**Table 1**; **Figure 3A**). Overexpression of *Sema-1a* resulted in α lobe overextension without affecting the sister β branches (**Table 1**; **Figures 3B,C**). The γ lobes appeared to overextend. These lobes had an irregular “accordion-like” anatomy, with axon tips that grew more ventrally instead of stopping at the midline. Both α and γ lobe phenotypes are opposite to the shorter lobes observed upon loss of *Sema-1a*. The β lobes showed overextension in 9% of the analyzed brains. However, it should be noted that in almost all the other brains analyzed, this phenotype could not be detected due to the complete absence of β lobes, as a result of the orientation phenotype (**Table 2**).

**Table 1:** Sema-1a affects lobe length.

	# Hemispheres	Lobe	% Overextension	% Short
*Sema-1a^CA07125^*	20	α	0	30
		β	35	0
		γ	0	0

*Sema-1a^K13702^/Sema-1a^CA07125^*	20	α	0	80
		β	0	5
		γ	0	10

*Sema-1a^ca07125^/Df(2L)Exel7039*	16	α	0	38
		β	19	6
		γ	0	50

UAS-*RNAi Sema-1a;;; OK107*-*Gal4*	14	α	0	21
		β	0	50
		γ	0	57

UAS-*Sema-1a;; OK107-Gal4*	44	α	100	0
		β	9	0
	30	γ	100	0

UAS-*Sema-1a*^Δ^*^cyt1^*; *OK107-Gal4*	22	α	0	0
		β	0	0
	42	γ	0	76

UAS-*Sema-1a^Δcyt2^*; *OK107-Gal4*	20	α	0	0
		β	0	10
		γ	0	100

**Table 2:** Sema-1a affects lobe orientation.

	# hemispheres	Lobe	% mis-orientation	
			Ventral-medial	Dorsal-lateral
*Sema-1a^CA07125^*	20	α	35	20
		β	25	0
		γ	0	5

*Sema-1a^K13702^/Sema-1a^CA07125^*	20	α	5	40
		β	30	0
		γ	0	10

*Sema-1a^ca07125^/Df(2L)Exel7039*	16	α	31	44
		β	25	0
		γ	0	0

UAS-*RNAi Sema-1a; OK107*-*Gal4*	14	α	0	0
		β	0	0
		γ	0	0

UAS-*Sema-1a*; *OK107-Gal4*	44	α	0	0
		β	0	84
	30	γ	0	0

UAS-*Sema-1a*^Δ^*^cyt1^*; *OK107-Gal4*	22	α	0	0
		β	0	4
	42	γ	0	0

UAS-*Sema-1a^Δcyt2^*; *OK107-Gal4*	20	α	0	0
		β	0	0
		γ	0	0

These data demonstrate that the effects of Sema-1a on α and γ lobe length are MB intrinsic with loss and gain of Sema-1a leading to shorter and longer α and γ lobes, respectively. The effects of Sema-1a on β lobe length, however, seem to be, at least partially, MB extrinsic. Analyzing homozygous *Sema-1a^k13702^*/*Sema-1a^k13702^* clones using MARCM partially confirmed these results, but also emphasized the complexity of the role of Sema-1a in MB development. While GFP labeled α and β lobe clones show length defects, surrounding unlabeled axons project normally (Supplementary Figures S2A–C). The short β lobe phenotype of the clones is consistent with the MB intrinsic effect observed upon RNAi knock-down. The fact that only the *Sema-1a* mutant clones show a phenotype in the α lobe confirms the MB intrinsic role of Sema-1a. However, contrary to the mutants and the RNAi knock-down where shorter lobes were observed, the MARCM mutant clones show overextension. We propose that these observations may reflect the need of Sema-1a levels to be carefully tuned to facilitate normal MB lobe formation or the presence of a Sema-1a modulated interaction between axons of the same lobe, or both. Previously, it has been reported that the TIFR can be involved in the regulation of midline crossing of horizontal MB lobes (Simon et al., 1998). The TIFR is a glial structure, located between the β lobes during late third instar and early pupal stages, when the β lobes are formed. Hence, we hypothesized that Sema-1a signaling in the TIFR could provide a ‘stop’ signal to growing β lobes. First, we checked whether *Sema-1a* is expressed in the TIFR. We labeled the TIFR by overexpressing *uas-mCD8-Rfp* using the TIFR-specific *422-Gal4* driver. Using the protein trap *Sema-1a^CA07125^*, we show that Sema-1a is located in the glia of the TIFR during early pupal stages (**Figure 3E**). To determine whether the midline crossing phenotype of the β lobes could be due to a role of Sema-1a in neuronal- glial communication during metamorphosis, we knocked down *Sema-1a* in glia using *repo-Gal4*. Pan-glial knock-down resulted in β lobe overextension and midline crossing (16 out of 22 hemispheres; *p* < 0.00001). Next, we analyzed whether this effect involves the glial TIFR cells. We made use of 442-*Gal4* to induce UAS-*Sema-1a*-RNAi and UAS-*Sema-1a* in the TIFR cells (**Table 3**; **Figure 3F**). Expression of both transgenes resulted in 100% penetrant β lobe overextension and fusion, while the γ lobes remained normal. This observation argues for a function of Sema-1a in the interhemispheric ring that is required for the correct development of the β lobes. Alternatively, TIFR-expressed *Sema-1a* may indirectly regulate β lobe formation by contributing to the development of the TIFR, whose wild-type structure might be crucial for inhibiting β lobe overextension. Analysis of the TIFR, however, showed no obvious morphological defects, indicating tha*t Sema-1a* does not have a crucial function in the development of this glial structure (Supplementary Figure S3). Furthermore, defective TIFR development has been shown to induce both β and γ lobe overextension while we observe only β defects (Simon et al., 1998). This supports a role for Sema-1a as a repulsive guidance cue in these glia rather than having a role in TIFR development. However, the fact that both knock-down and overexpression of *Sema-1a* in the TIFR result in β-lobe overextension suggests that Sema-1a in the TIFR may influence β-lobe development in more than one way.

**Table 3:** Sema-1a is required in the glial interhemispheric ring.

	# hemispheres	Lobe	% overextension
UAS-RNAi-*Sema-1a; 442*-*Gal4*	18	α	0
		β	100
UAS-*Sema-1a; 442*-*Gal4*	20	α	0
		β	100
UAS-*Sema-1a^Δcyt1^; 442*-*Gal4*	20	α	0
		β	0
UAS-RNAi-*PlexA; 442*-*Gal4*	26	α	0
		β	0
UAS-RNAi-*PlexB; 442*-*Gal4*	20	α	0
		β	0

Sema-1a has previously been shown to signal in a bidirectional manner, resulting in both a ligand and a receptor function for the protein (Godenschwege et al., 2002). To investigate these signaling modalities in the context of the MBs, we made use of overexpression constructs containing both the transmembrane and extracellular regions of the native protein but lacking the complete intracellular domain (*Sema-1a^Δcyt1^* and *Sema-1a^Δcyt2^*; Godenschwege et al., 2002). It was previously shown that the cytosolic fragment is not required for Sema-1a surface expression, as Sema-1a^Δcyt1^ is highly expressed in MB axons and is functionally active as a cell-surface ligand in the giant fiber system (Godenschwege et al., 2002; Komiyama et al., 2007). The α and γ lobe overextension phenotypes, induced with overexpression of full-length Sema-1a, were completely absent in *Sema-1a^Δcyt1^*-overexpressing MBs (**Table 1**). Instead, the α lobes had a wild type morphology, while most of the γ axons were shorter than in wild type controls (**Figure 3D**). Similar results were obtained with a second independently generated line, *Sema-1a^Δcyt2^* (**Table 1**). These data suggest that the cytoplasmic portion of Sema-1a could be required to provide MB axons with an outgrowth signal, and thus that Sema-1a acts as an outside-in receptor in lobe outgrowth. Alternatively, the cytoplasmic domain of the Sema-1a protein could also play a regulatory role for Sema-1a as a ligand, such as fine-tuning of ligand levels or regulation of subcellular and compartment localization. Next we overexpressed *Sema-1a^Δcyt1^* in the glia of the TIFR using *442-Gal4*. While overexpression of full length *Sema-1a* led to β lobe overextension, this phenotype is absent upon overexpression of the truncated protein (**Table 3**). These data indicate that the cytoplasmic portion of Sema-1a in the TIFR glia is required to regulate MB β lobe axon extension, and that Sema-1a could also act as a receptor in addition to acting as a ligand as suggested by the β-lobe overextension phenotype seen upon knock-down of Sema-1a in the TIFR. A receptor function of Sema-1a in glia would require additional pathways or downstream mechanisms which signal back to the β lobe axons to regulate their outgrowth. Alternatively, as suggested above, the cytoplasmic domain of the Sema-1a protein could play a regulatory role for Sema-1a as a ligand. In summary, we show that Sema-1a affects lobe outgrowth and has axonal sister branch-specific effects. Although, α and β lobes derive from the same axon, these neurites respond differentially to alterations in Sema-1a signaling. Furthermore, while the effects on the other MB lobes appear to be MB intrinsic, β lobe outgrowth appears regulated by both MB intrinsic and extrinsic Sema-1a signaling. Our data suggests a role for Sema-1a in the glia of the TIFR in this process. Finally, we show that the cytoplasmic portion of Sema-1a modulates α and γ lobe overextension.

### *Sema-1a* has MB Extrinsic Effects on Lobe Orientation

*Sema-1a^CA07125^, Sema-1a^k13702^*/*Sema-1a^CA07125^* and *Sema-1a^k13702^*/*Df(2l)Exel7039 trans*-heterozygotes also show a second range of phenotypes. Loss of Sema-1a results in misorientation of the MB lobes (**Table 2**; **Figure 2**). As observed with the effects of Sema-1a on lobe length, the effects on lobe orientation are also sister-branch specific. The misorientation defects observed comprised the α lobes growing more dorso-laterally than normal, or more ventro-medially resulting in growth parallel to the β lobes (**Figures 2A,B**). The β lobes on the other hand grew more ventrally than in wild type control brains (**Figure 2A**). The γ lobes showed dorso-lateral misorientation in a limited number of brains (**Table 2**).

To determine whether Sema-1a is required MB intrinsically for lobe orientation, we looked at the effects of *Sema-1a* RNAi-mediated knock-down using the *OK107-Gal4* MB-driver. Knock-down of *Sema-1a* in the MBs had no effect on lobe orientation (**Table 2**). Hence, we conclude that the effects of Sema-1a on MB orientation are MB extrinsic. This was confirmed by analyzing homozygous *Sema-1a^k13702^*/*Sema-1a^k13702^* clones using MARCM analyses. Both GFP labeled clones and unlabeled axons misorient (Supplementary Figure S2D). Although, MB intrinsic Sema-1a does not seem to be required for lobe orientation during normal development, overexpression of Sema-1a can influence β lobe orientation (**Table 2**). This effect seems to involve the Sema-1a cytoplasmic domain which might suggest a receptor function of the protein. This shows the requirement of complex fine tuning of Sema-1a in different cell types during development.

In summary, Sema-1a affects MB lobe length and orientation in a lobe and lobe sister branch-specific manner. While lobe length involves both MB intrinsic and extrinsic Sema-1a signaling, our data suggest that lobe orientation mainly depends on MB lobe extrinsic Sema-1a signaling. However, further experiments will be required to elucidate the exact mechanisms by which Sema-1a acts and whether it acts as a ligand or receptor involving only other MB axons or also other neuronal or non-neuronal cells. Due to the complexity of the observed phenotypes, the requirement of subtle fine tuning of this pathway and the broad expression of *Sema-1a* in the brain, correct MB development most likely relies on the integration of the combined effects of Sema-1a and the various possible mechanisms and interactions that seem involved.

### PlexA and PlexB are Involved in MB Development and Interact with Sema-1a

We showed a complex role for Sema-1a in both MB lobe outgrowth and orientation. Plexins are well-characterized Semaphorin receptors, involved in both forward and reverse Semaphorin signaling (Whitford and Ghosh, 2001; Yu et al., 2010). In *Drosophila*, PlexA has been shown to function as a Sema-1a and Sema-1b receptor, while both *PlexA* and *PlexB* have been shown to genetically interact with the secreted Sema-2a protein (Winberg et al., 1998; Ayoob et al., 2006; Bates and Whitington, 2007). *PlexA* has been indirectly implicated in αβ lobe development through interactions with *Highwire* and *off-track* (Shimizu et al., 2011; Shin and DiAntonio, 2011). Very little is known about the expression of both Plexins in the *Drosophila* nervous system. By means of *in situ* hybridization, we show *PlexA* and *PlexB* expression in the embryonic central nervous system in concordance with previously described data (Terman et al., 2002; Ayoob et al., 2006; Supplementary Figures S4A,B). Furthermore, using the *PlexA^Y D0269^* GFP reporter trap line, we show *PlexA* expression in the adult MB (Supplementary Figure S4C). Next, we took a closer look at the possible roles of PlexA and PlexB in MB formation.

**FIGURE 4:**
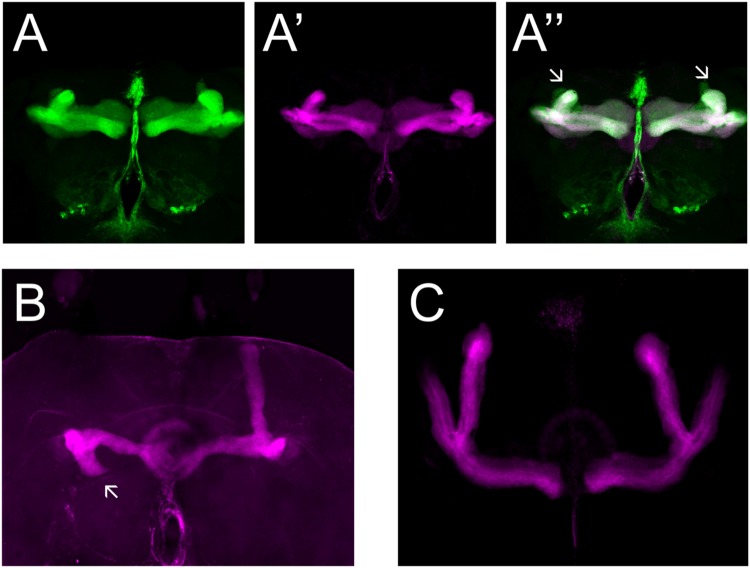
**PlexA and PlexB are involved in MB development. (A)** *UAS-RNAi-PlexA;UAS-mCD8-Gfp;OK107-Gal4*, **(A)** anti-FasII staining (magenta), **(A′)** GFP (green), **(A′′)** overlay. The α lobes show outgrowth defects (arrows). **(B)** *PlexB^KG0087^*, anti-FasII staining (magenta). One α lobe is shorter and shows misorientation (arrow). **(C)** *UAS-RNAi-PlexB;;OK107-Gal4*, anti-FasII staining. Knock-down of *PlexB* in the MB causes no neuroanatomical MB defects.

We made use of two mutant alleles (*PlexA^EY 16548^* and *PlexB^KG00878^*), as well as RNAi constructs targeting each gene (Hu et al., 2001; Sweeney et al., 2007; Yu et al., 2010). As previously reported, heterozygous *PlexA^EY 16548^* flies (homozygous lethal) showed no obvious MB abnormalities (**Table 4**; Shin and DiAntonio, 2011). However, RNAi-mediated knock-down of this gene using *OK107-Gal4* led to strong defects in α, β and γ lobe outgrowth (**Table 4**; **Figure 4A**). In some cases, β lobe misguidance was observed (**Table 4**). *PlexB^KG0087^* homozygotes are semi-lethal and survivors had short α, β, and γ lobes (**Figure 4B**). Furthermore, these lobes also showed misorientation phenotypes. Some α lobes grew parallel to the β lobes, while some of these lobes grew more ventrally. Misoriented γ lobes grew more dorsally (**Table 4**). These phenotypes are remarkably similar to the ones seen in *Sema-1a* loss of function mutants. *OK107-Gal4*-driven RNAi knock-down of *PlexB* caused no neuroanatomical MB defects suggesting that PlexB functions in cells extrinsic to MBs (**Table 4**; **Figure 4C**). Our data mainly implicate a role for PlexA in stimulating outgrowth in the MB neurons, while PlexB seems to be involved in both outgrowth and orientation.

**Table 4:** PlexA and PlexB are involved in MB development.

	# hemispheres	Lobe	% short	% misorientation	
				Ventral-medial	Dorsal-lateral
*PlexA^EY 16548^/+*	20	α	0	0	0
		β	0	0	0
		γ	0	0	0

UAS-RNAi-*PlexA*; *OK107-Gal4*	20	α	50	0	0
		β	20	0	15
		γ	20	0	0

*PlexB^KG00878^*	14	α	7	14	14
		β	14	43	0
		γ	36	0	21

UAS-RNAi-*PlexB*; *OK107-Gal4*	20	α	0	0	0
		β	0	0	0
		γ	0	0	0

PlexA is known to be a Sema-1a ligand and has been shown to modulate reverse Sema-1a signaling in photoreceptor axon guidance (Yu et al., 2010). Hence, we wanted to investigate which effects of Sema-1a signaling in the MBs depend on PlexA. PlexB has not been implicated in MB development before, nor has it been shown to interact with Sema-1a. Introduction of *PlexA^EY 16548^* or *PlexB^KG00878^* alleles in *Sema-1a^k13702^/Sema-1a^CA07125^ trans*-heterozygotes resulted in a profound phenotypic shift (**Table 5**). Both alleles caused a reduction or loss of the α lobe outgrowth defect (*p* < 0.0001), while β misorientation defects remained unaffected. We observed no effects on γ lobe morphology. *PlexB^KG00878^* introduction also led to a significant increase in ventral medial α lobe misguidance (*p* = 0.0385). These data confirm our findings that PlexA is mainly involved in lobe outgrowth while PlexB influences both outgrowth and orientation. We confirm the genetic interaction between *PlexA* and *PlexB* and *Sema-1a* combining either a *PlexA^EY 16548^* or *PlexB^KG00878^* allele (or an RNAi construct against one of these receptors) with *OK107-Gal4*-driven *Sema-1a* overexpression. Introduction of these alleles led to a robust decrease in α overextension (**Table 6**). However, the correctly guided β axons failed to stop at the midline. This finding is consistent with our previous results showing a MB extrinsic effect of Sema-1a in the TIFR glia. We previously showed that MB intrinsic Sema-1a signaling is not required during development for correct lobe orientation, but overexpressed Sema-1a can influence this phenotype. We show that both PlexA and PlexB influence the effects of Sema-1a overexpression on lobe orientation, although the latter does not seem to be required for correct lobe orientation during development (Supplementary Table S4). These findings suggest a complex interaction between PlexA and PlexB with Sema-1a, which modulates different sister-branch specific effects in the different MB lobes. The complexity of this interaction is further emphasized by the suppression of the effect on α lobe outgrowth upon loss and overexpression of *Sema-1a*. Hence, the role of Sema-1a in the development of the MB most likely depends on careful fine tuning of MB intrinsic and extrinsic effects involving different MB axons and cell types, modulation by different receptors and interactions with other developmental pathways. Finally, we checked whether PlexA or PlexB were also involved in the regulation of β lobe overextension in the TIFR glia. Knock-down of both Plexins in this structure using *442-Gal4* had no effect on MB lobe morphology (**Table 3**).

**Table 5:** *PlexA* and *PlexB* show genetic interaction with Sema-1a trans-heterozygous mutants (Fisher exact test: *p < 0.05, ****p < 0.0001).

	# hemispheres	Lobe	% short	% misorientation	
				ventral-medial	dorsal-lateral
*Sema-1a^K13702^/Sema-1a^CA07125^*	20	α	80	5	40
		β	5	30	0
		γ	10	0	0

*Sema-1a^K13702^/Sema-1a^CA07125^; PlexA^ey16548^/+*	24	α	10****	5	29
		β	5	17	0
		γ	0	0	0

*Sema-1a^K13702^/Sema-1a^CA^; PlexB^kg00878^/+*	24	α	0****	29*	25
		β	0	13	4
		γ	0	0	0

**Table 6:** PlexA and PlexB show genetic interaction with Sema-1a overexpression (Fisher exact test: *p < 0.05, ****p < 0.0001).

	# hemispheres	Lobe	% overextension
UAS-*Sema-1a*; *OK107-Gal4*	44	α	100
		β	9
UAS-*Sema-1a*; *PlexA^ey16548^/+*; *OK107-Gal4*	48	α	54****


		β	65****
UAS-*Sema-1a*; *PlexB^kg00878^/+*; *OK107-Gal4*	20	α	65****


		β	65****
UAS-*Sema-1a*/UAS-RNAi-*PlexA; OK107-Gal4*	22	α	86*


		β	0
UAS-*Sema-1a*/UAS-RNAi-*PlexB; OK107-Gal4*	26	α	92
		β	29*

## Discussion

Our findings show that *Sema-1a* is expressed in MB lobes throughout development and that Sema-1a signaling sculpts MB axon morphology in a lobe and axon branch-specific manner. Sema-1a seems to direct both outgrowth, resulting in either shorter or overextended lobes, and orientation, in which Sema-1a is responsible for the angle in which the individual lobes grow.

Mushroom body lobe-specific effects have been previously reported for other guidance cues, including *Neuroglian* and *Highwire*. Interestingly, these genes have both been reported to interact with *Sema-1a* or *PlexA*, respectively (Godenschwege and Murphey, 2009; Goossens et al., 2011; Shin and DiAntonio, 2011).

Mutations in *Highwire* only affect αβ lobe development and result in phenotypes that are remarkably similar to a subset of the phenotypes that we observed in *Sema-1a* mutants (Shin and DiAntonio, 2011). *Highwire* loss of function most often results in β axons growing upward alongside the α axons, although in some cases the opposite effect is observed. Furthermore, these authors observed an interaction between *Highwire* and *PlexA*, but no genetic interaction was observed with *Sema-1a*. In addition, they reported no obvious effects on MB development in both heterozygous *Sema-1a^k13703^* mutants and *OK107-Gal4*-driven RNAi knock-down. However, given our observations regarding the role of Sema-1a in MB development, the presence of a partial orientation phenotype in *Highwire* mutants and an interaction between this gene and *PlexA*, it is tempting to speculate that *Highwire* could be involved as a component of the *Sema-1a* pathway responsible for modulating specific αβ growth. As Highwire is a ubiquitin ligase it could directly modulate PlexA degradation, or it could indirectly interact with PlexA via interaction with the Wallenda MAP kinase kinase kinase, as previously suggested (Wu et al., 2007; Shin and DiAntonio, 2011).

Axon branch formation has been well-studied and shown to involve different processes, including localized protein synthesis and calcium transients (Hutchins and Kalil, 2008; Spillane et al., 2012). Different axon guidance cues have been shown to be involved in these branching processes, including Semaphorin-3A and Plexin-A3 in vertebrates and PlexA and PlexB in *Drosophila* (Neufeld et al., 2011; Sainath and Granato, 2013). However, which mechanisms are responsible for the subsequent differential guidance of these newly formed axonal branches is largely unknown. While the growth of axons in a different direction than dendrites in response to the same guidance cue has frequently been described (e.g., Polleux et al., 2000), the mechanisms that enable axonal sister branches to extend in different directions during development are poorly understood. Strikingly, one of the few well-documented examples of differential axonal responsiveness to single guidance cues involves Semaphorin-3D (Liu and Halloran, 2005). Our data suggest that different Sema-1a signaling pathways are involved in the guidance of axon branches that derive from the same neuron. In each of our Sema-1a loss-of-function analyses, gain-of-function experiments and genetic interaction tests, we observed distinct phenotypes in the horizontal versus vertical lobes.

Besides an effect of Sema-1a in MB neurons, we also show that this protein functions in the glial cells of the TIFR to regulate β lobe extension during metamorphosis. Neuron-glia communication involving axon guidance molecules has previously been shown to play important roles in embryonic nervous system development (Lemke, 2001). For example, Netrin and slit secreted by midline glia regulate commissural crossing at the neural midline in the *Drosophila* embryo. Glia have also been shown to be involved in the remodeling of the brain during metamorphosis. They are essential for the pruning of MB axons as well as for the removal of neuronal debris caused by the intense remodeling of the brain (Cantera and Technau, 1996; Awasaki and Ito, 2004; Parker and Auld, 2006). However, very little is known about the role of glia in axon guidance during metamorphosis. TIFR glia cells have been previously shown to play an important role in midline crossing of olfactory receptor neurons in the antennal lobe, as well as in midline crossing of MB β and γ lobes. However, these phenotypes seem to be most likely due to improper development of the TIFR (Simon et al., 1998; Chen and Hing, 2008). Our data provides evidence for a guidance role of TIFR Sema-1a in β lobe development, which is independent of a role of this protein in TIFR development. Interestingly, neuronal-glial communication involving vertebrate Semaphorin-6D and Nr-CAM/Plexin-A1 has been previously shown to modulate the midline crossing of retinal ganglion cells at the optic chiasm in mice (Kuwajima et al., 2012).

In *Drosophila*, Sema-1a has also been shown to function as a receptor during development of the α′β′ MB lobes, the giant fiber system, the adult photoreceptor axons, the dendrites of the projection neurons in the olfactory system and the embryonic motor neurons (Godenschwege et al., 2002; Cafferty et al., 2006; Komiyama et al., 2007; Jeong et al., 2012). Exploring Sema-1a signaling mechanisms using full-length and truncated overexpression constructs, we now show that Sema-1a also plays a cell autonomous role in the MBs. Our data point to an important regulatory role of the cytoplasmic portion of Sema-1a in both lobe orientation and outgrowth.

Sema-1a was previously shown to signal via PlexA. This interaction can lead to bidirectional signaling, which involves Rho GTPases downstream of both Sema-1a and PlexA and Off-track downstream of PlexA (Driessens et al., 2001; Hu et al., 2001; Yu et al., 2010). Furthermore, PlexA has been indirectly shown to be involved in αβ lobe development (Shimizu et al., 2011; Shin and DiAntonio, 2011). PlexB has not been previously shown to be involved in MB development or Sema-1a signaling. Here we confirm the role of PlexA in MB development and Sema-1a signaling and provide evidence of a genetic interaction between PlexB and Sema-1a in MB development.

The complex genetic interactions that we observe between Sema-1a and its different interaction partners could be attributed to various processes. Our data show a complex involvement of Sema-1a in MB development regulated by interactions with different receptors. The correct integration of these signals seems to provide lobe and sister branch- specific signals leading to correct MB lobe outgrowth and orientation. Furthermore, Sema-1a signaling also depends on the integration of signals from multiple cell types, including neuron-glia interactions. Finally, it has been shown that multiple regulatory mechanisms can underlie Semaphorin/Plexin signaling, resulting in a context dependent role of these pathways. Vertebrate Plexin-A1/Sema-3a signaling, for instance, is modulated by auto inhibition of Plexin-A1 by its own Sema domain, while interactions *in trans* between Sema-6A and Plexin-A4 have been shown to modulate *cis* interaction between these molecules during axon guidance (Takahashi and Strittmatter, 2001; Haklai-Topper et al., 2010). Furthermore, in *Drosophila* it has been proposed that different GTPase modulators contribute to the context dependent integration of parallel PlexA/Sema-1a and Sema-1a reverse signaling (Jeong et al., 2012).

In summary, we provide evidence for a complex role of Sema-1a signaling in MB development. Sema-1a seems to function via different mechanisms to provide tightly regulated outgrowth and directional cues to the different lobes and sister-branches. In both these processes, Sema-1a plays a cell-autonomous role in the MB neurons, but it is also involved in non-cell autonomous neuron-glia signaling.

## Author Contributions

LZ, TG, YK, and JC designed and performed the experiments, and wrote the manuscript. PC designed and supervised the project and assisted in data interpretation and writing the manuscript.

## Conflict of Interest Statement

The authors declare that the research was conducted in the absence of any commercial or financial relationships that could be construed as a potential conflict of interest.
