# Supplementary material for: Axon Branch-Specific Semaphorin-1a Signaling in Drosophila Mushroom Body Development
Source: Front Cell Neurosci. 2016 Sep 5;10:210. doi: 10.3389/fncel.2016.00210 (PMC5011136; doi:10.3389/fncel.2016.00210)
Supplement: Supplementary file 1 [file Data_Sheet_1.PDF]

Supplementary Material

Axon branch-specific *Semaphorin-1a* signaling in *Drosophila* mushroom body development

L Zwarts*, T Goossens, J Clements, YY Kang, P Callaerts*

*** Correspondence:** patrick.callaerts@kuleuven.be, liesbeth.zwarts@kuleuven

1 Supplementary Figures

1.1.1 Supplementary Figure S1: Sema-1a expression analysis

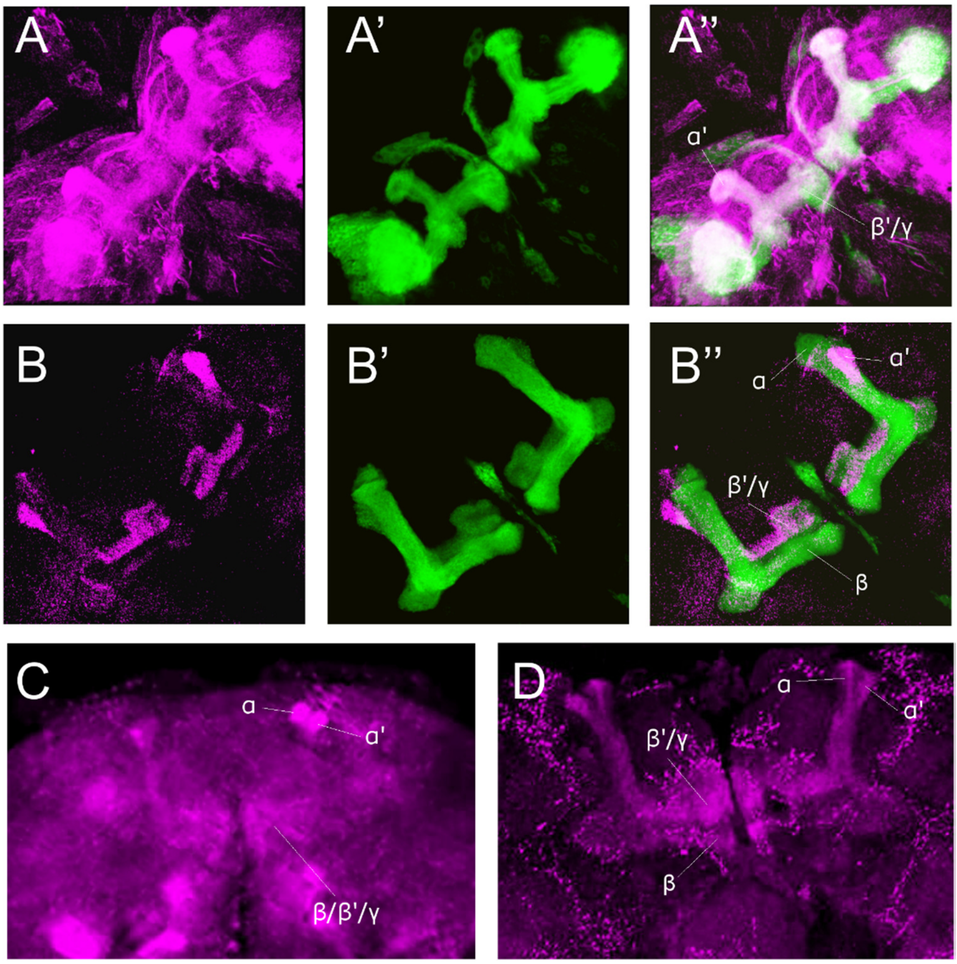

A. Sema-1a MB expression in 3rd instar larvae: *OK107-Gal4*-driven UAS-*mCD8-Gfp* (green), Anti-Sema-1a staining (magenta). B. Sema-1a MB expression in 50% pupae: *OK107-Gal4*-driven UAS-*mCD8-Gfp* (green), Anti-Sema-1a staining (magenta). Sema-1a is MB expression in 50% pupae: Anti-Sema-1a staining (magenta). C. Sema-1a MB expression in 75% pupae. D. Sema-1a MB expression in adults: anti-Sema-1a staining (magenta).

1.1.2 **Supplementary Figure S2: MARCM analysis**

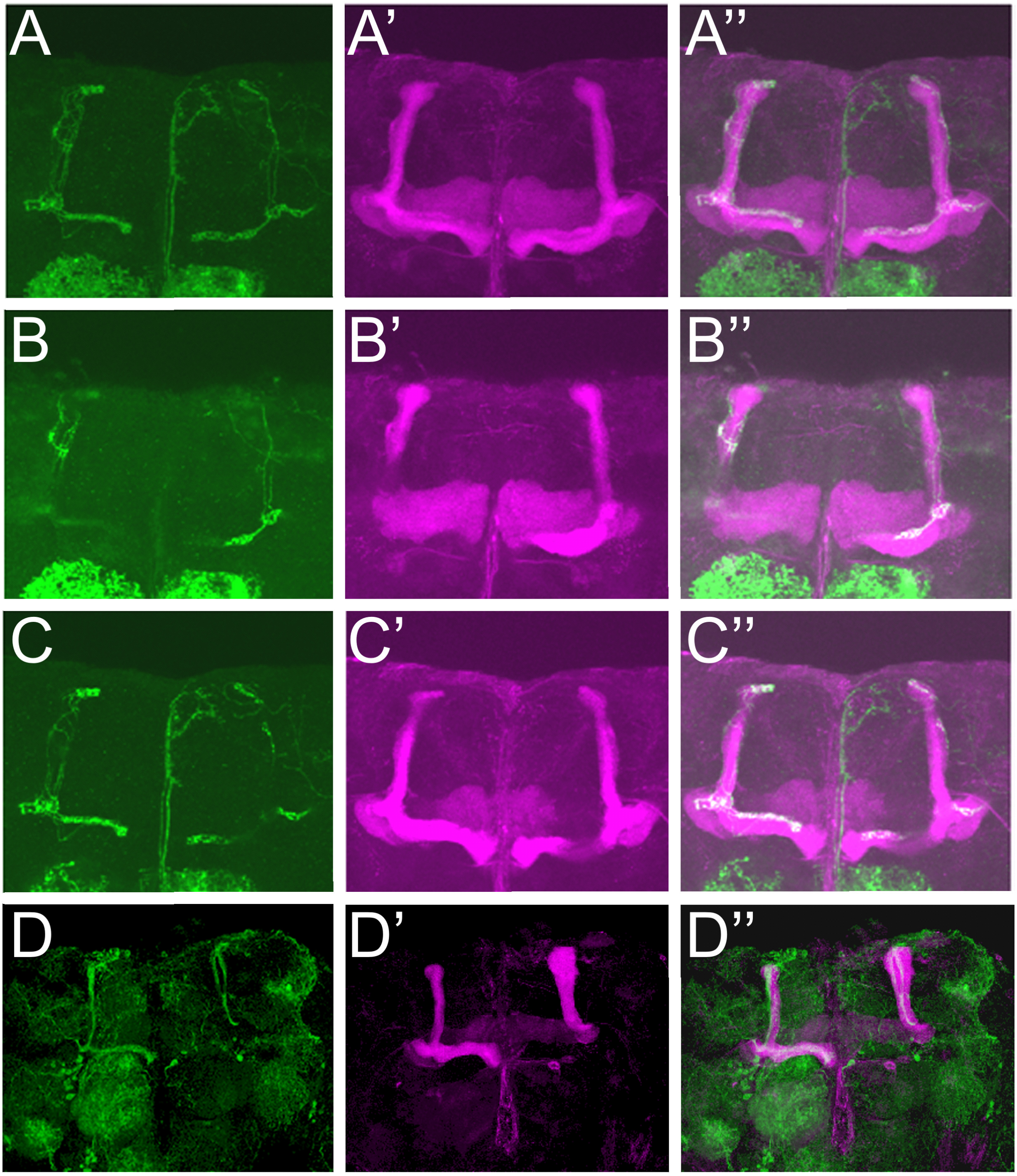

hsFLP; *Sema-1a*^{k13702} *FRT40a*/ *FRT40A-Gal80; Tub-Gal4,* UAS-*mCD8-Gfp*. A-C. *Sema-1a*^{k13702}/ *Sema-1a*^{k13702} neurons (UAS-*mCD8-Gfp*; green) show length defects. Surrounding heterozygous *Sema-1a*^{k13702} neurons (anti-FasII; magenta) project normally. A-C represent different confocal sections (N= 5). D. Both *Sema-1a*^{k13702}/ *Sema-1a*^{k13702} clones (UAS-*mCD8-Gfp*; green) and surrounding heterozygous *Sema-1a*^{k13702} (anti-FasII; magenta) neurons misorientate (N=5).

1.1.3 Supplementary Figure S3: Overexpressing *UAS-Sema-1a* or *UAS-RNAi-Sema-1a* in the TIFR results in no obvious morphological defects

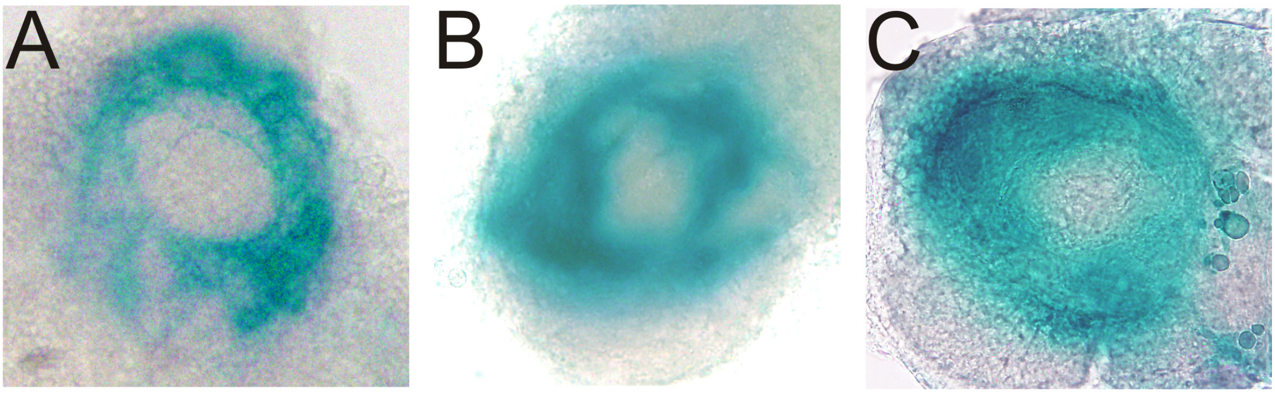

(A-C) β-galactosidase staining of TIFR's of pupal brains 48 hours APF (N=5). (A) UAS-*lacZ.btau.YES; 442-Gal4* (B) UAS-RNAi-*Sema-1a*; UAS-*lacZ.btau.YES; 442-Gal4* (C) UAS-*Sema-1a*/ UAS-*lacZ.btau.YES; 442-Gal4*.

1.1.4 Supplementary Figure S4: *PlexA* and *PlexB* expression in the ventral nerve cord in the embryo and *PlexA* expression in the adult MB

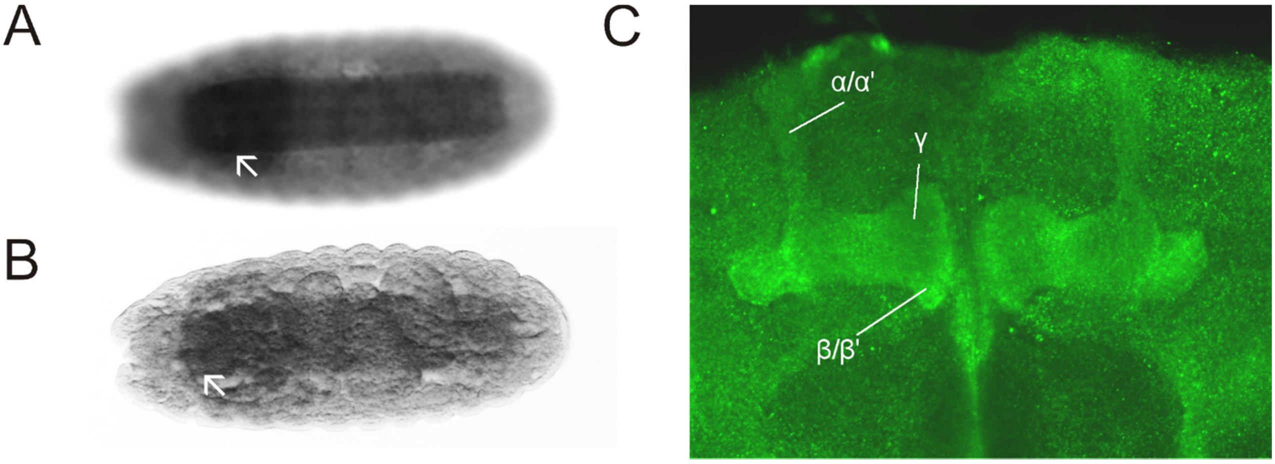

(A-B) *In situ* hybridization showing expression in the embryonal central nervous system (arrows). (A) *PlexA* expression. (B) *PlexB* expression. (C) $PlexA^{YD0269}$: The GFP reporter trap line shows expression in the MB lobes in the adult brain.

2 Supplementary Tables

2.1.1 Supplementary table S1: Sema-1a affects α 'and β ' lobe length

In *Sema-1a*^{CA07125} homozygotes, and *Sema-1a*^{k13702}/ *Sema-1a*^{CA07125} and *Sema-1a*^{k13702}/ *Df(2l)Exel7039* double heterozygote flies, we could observe outgrowth defects in α' and β' resulting in short lobes. Knock-down of *Sema-1a* in the MB using *OK107-Gal4* resulted in shorter α'β' lobes, similar to the phenotypes observed in the mutants. Thus, the effect of Sema-1a on the length of these lobes seems to be MB intrinsic. Overexpression of the truncated *Sema-1a*$^{\Delta cyt}$ construct resulted in α' lobes with normal lobe lengths, while the β' lobes were often short (*UAS-Sema-1a*$^{\Delta cyt1}$;; *OK107-Gal4*: 45%; *UAS-Sema-1a*$^{\Delta cyt2}$; *OK107-Gal4*: 15%). This suggests that Sema-1a could act as a receptor to modulate α' and β' lobe overextension.

	# hemispheres	lobe	% overextension	% short
Sema-1a^{CA07125}	20	α'	0	30
		β'	0	10
Sema-1a^{K13702}/ *Sema-1a*^{CA07125}	20	α'	0	10
		β'	0	10
Sema-1a^{ca07125}/ *Df(2L)Exel7039*	16	α'	0	0
		β'	0	0
UAS-*RNAi Sema-1a;;;* OK107-*Gal4*	14	α'	0	21
		β'	0	57
UAS-*Sema-1a*;; *OK107-Gal4*	30	α'	0	0
		β'	0	0
UAS-*Sema-1a*$^{\Delta cyt1}$;; *OK107-Gal4*	42	α'	0	0
		β'	0	45
UAS-*Sema-1a*$^{\Delta cyt2}$; *OK107-Gal4*	20	α'	0	0
		β'	0	15

2.1.2 **Supplementary table S2: Sema-1a affects α'and β' lobe orientation**

In *Sema-1a*^{CA07125} homozygotes, and *Sema-1a*^{k13702}*/ Sema-1a*^{CA07125} and *Sema-1a*^{k13702}*/ Df(2l)Exel7039* double heterozygote flies we could observe some misorientation of the α' involving a more dorso-lateral orientation of the lobes. To examine the MB intrinsic requirement for Sema-1a on α' and β' lobe orientation, we looked at the effects of *Sema-1a* RNAi-mediated gene knock-down using the *OK107-Gal4* MB-driver. Knock-down of *Sema-1a* in the MBs had no effect on lobe orientation. Although MB intrinsic Sema-1a does not seem to be required for α' and β' lobe orientation during normal development, overexpression of *Sema-1a* can influence lobe orientation. Overexpression of *Sema-1a* in the MB using *OK107-Gal4* causes α' and β' lobes to bend towards each other and eventually fuse at their tips. This effect relies on the Sema-1a cytoplasmic domain. This shows the requirement of complex fine tuning of Sema-1a in different cell-types during development.

	**# hemispheres**	**lobe**	**% misorientation**	
			**ventral- medial**	**dorsal- lateral**
Sema-1a^{CA07125}	20	α'	0	40
		β'	0	0
Sema-1a^{K13702}*/ Sema-1a*^{CA07125}	20	α'	10	20
		β'	0	0
Sema-1a^{ca07125}*/ Df(2L)Exel7039*	16	α'	0	50
		β'	0	0
UAS-*RNAi-Sema-1a;;; OK107-Gal4*	14	α'	0	0
		β'	0	0
UAS-Sema-1a;; OK107-Gal4	30	α'	80	0
		β'	0	80
UAS-*Sema-1a*$^{\Delta cyt1}$;; *OK107-Gal4*	42	α'	0	0
		β'	0	0
UAS-*Sema-1a*$^{\Delta cyt2}$; *OK107-Gal4*	20	α'	0	0
		β'	0	0

2.1.3 **Supplementary table S3: PlexA and PlexB are involved in α'and β' development**

Knock-down of *PlexA* resulted in short lobes in 50% of the examined lobes.

	# hemispheres	lobe	% short	% misorientation	
				ventral-medial	dorsal-lateral
UAS-RNAi-*PlexA*;; *OK107-Gal4*	20	α'	50	0	0
		β'	0	0	0
UAS-RNAi *PlexB*;; *OK107-Gal4*	20	α'	0	0	0
		β'	0	0	0

2.1.4 Supplementary table S4: *PlexA* and *PlexB* show genetic interaction with *Sema-1a* overexpression (lobe orientation)

Fisher exact test: * p<0.05, **** P< 0.0001

	# hemispheres	lobe	% misorientation	
			ventral-medial	dorsal-lateral
UAS-*Sema-1a*;; *OK107-Gal4*	44	α	0	0
		β	0	84
UAS-*Sema-1a*; *PlexA*^{ey16548}/+; *OK107-Gal4*	48	α	0	11
		β	0	23****
UAS-*Sema-1a*; *PlexB*^{kg00878}/+; *OK107-Gal4*	20	α	0	0
		β	0	25****
UAS-*Sema-1a/* UAS RNAi *PlexA; OK107-Gal4*	22	α	0	45****
		β	0	100
UAS-*Sema-1a/* UAS RNAi *PlexB; OK107-Gal4*	26	α	18**	11*
		β	0	63*
